# Placebo and nocebo effects: from observation to harnessing and clinical application

**DOI:** 10.1038/s41398-022-02293-2

**Published:** 2022-12-24

**Authors:** Yiheng Tu, Libo Zhang, Jian Kong

**Affiliations:** 1 CAS Key Laboratory of Mental Health, Institute of Psychology, Chinese Academy of Sciences, Beijing, China; 2 Department of Psychology, University of Chinese Academy of Sciences, Beijing, China; 3 Department of Psychiatry, Massachusetts General Hospital and Harvard Medical School, Charlestown, MA USA

**Keywords:** Predictive markers, Psychiatric disorders

## Abstract

Placebo and nocebo effects are salubrious benefits and negative outcomes attributable to non-specific symbolic components. Leveraging advanced experimental and analytical approaches, recent studies have elucidated complicated neural mechanisms that may serve as a solid basis for harnessing the powerful self-healing and self-harming capacities and applying these findings to improve medical practice and minimize the unintended exacerbation of symptoms in medical practice. We review advances in employing psychosocial, pharmacological, and neuromodulation approaches to modulate/harness placebo and nocebo effects. While these approaches show promising potential, translating these research findings into clinical settings still requires careful methodological, technical, and ethical considerations.

## Introduction

Placebo and nocebo effects are essential components of clinical practice and efficacy research [1]. They occur in both experimental and clinical contexts when a pure inert treatment is administered on its own or as part of active treatments. A significant proportion of clinical improvement, particularly the subjective symptom relief, may be attributable to placebo effects [2, 3]. In contrast, nocebo effects are a major concern for clinical care since patients are often non-compliant, make unnecessary medical visits, and take additional medications to counteract adverse effects that are actually nocebo effects [4–6]. Placebo and nocebo effects have been observed in a plethora of conditions including pain, Parkinson’s disease, depression, anxiety disorders, immunologic responses, cardiovascular functions, and sleep disorders [7].

In recent years, considerable efforts have been made to conceptualize placebo and nocebo effects in a broad variety of disciplines from clinical sciences to cognitive neuroscience and social sciences. The improved understanding of placebo and nocebo effects has built a basis for the crucial next step: shifting from understanding their biopsychosocial mechanisms through systematic observation to modulating placebo and nocebo effects through experimental paradigms/designs or brain stimulation methods. The proposed research focus shift echoes the growing interest in optimizing placebo effects to improve therapeutic outcomes and minimizing nocebo effects to avoid unintended exacerbation of symptoms in medical practice.

This review discusses recent advances in placebo and nocebo research in moving from observation to experimental mechanistic modulation and finally to clinical practice. We first briefly survey key mechanisms involved in the placebo and nocebo effects. Based on these mechanisms, we then discuss recent attempts at modulating these effects using psychosocial, pharmacological, and neuromodulation approaches. Finally, we discuss approaches and challenges to harness these effects ethically and effectively in clinical settings. We will focus on placebo analgesia and nocebo hyperalgesia as the majority of placebo and nocebo research centers on pain; we will also highlight mechanistic heterogeneity of placebo and nocebo effects in other domains/conditions as appropriate.

## Behavioral and neural bases for harnessing placebo and nocebo effects

### Behavioral bases of placebo and nocebo effects

Expectations and learning are two frequently studied behavioral mechanisms associated with placebo and nocebo effects [1, 8]. Numerous studies have demonstrated that expectations of receiving treatment induce placebo and nocebo effects [9, 10]. Expectations can be generated through verbal information [11, 12], which involves the provision of direct information about the efficacy of treatment. Alternatively, expectations can be created by associative learning, especially classic conditioning, which repetitively pairs a neutral cue with an active treatment [13]. Conditioning-based expectations have been shown to exert strong effects on pain (i.e., placebo analgesia and nocebo hyperalgesia) [14–21] and other subjective symptoms including emotion, Parkinson’s disease, and depression [22].

Aside from learning from direct experience (i.e., classic conditioning), observational conditioning may also produce placebo and nocebo effects. Studies have demonstrated that participants will feel less pain after receiving treatment if they see this treatment is effective in others [23–25]. Similarly, cue-based expectations can be learned from observing others’ painful experiences, and in turn modulate subsequent subjective perception [15, 26]. Although most of these studies have focused on the reduction of pain, the effects of social learning may be generalized to other modalities [27], for example, psychogenic illness [28, 29]. Therefore, social learning is a major routine for the transmission of placebo/nocebo effects and produces substantial effects on the associated brain processes.

Recent placebo research showed operant conditioning as a new mechanism of placebo effects [30]. In operant conditioning, responses, instead of cues, are reinforced. In a recent study [31], participants’ pain ratings of identical electrocutaneous pain stimuli preceded by visual cues were either verbally rewarded or punished. Placebo analgesia was successfully established by rewarding low ratings and punishing high ratings, and, interestingly, seemed resistant to extinction. Another study revealed that operant conditioning generated greater placebo analgesia than classical conditioning and that mechanical pain-induced brain activities in the ipsilateral S1 and contralateral lingual gyrus were reduced more by operant conditioning [32]. These findings revealed that patients can learn placebo analgesia as a result of operant learning. Altogether, these studies suggest that expectations can be finely tuned by different forms of learning and, thus, may provide flexible and alternative ways to induce placebos in medical practice.

Apart from expectations and learning, other behavioral bases of placebo and nocebo effects have also been proposed. For example, the desire for pain relief has been suggested as a key contributor to placebo analgesia [33]. In an early study, combined with expectations, the desire for pain relief explained approximately 80% of the variance of placebo analgesia in irritable bowel syndrome patients [34]. However, perhaps due to limited sample sizes and specific painful stimuli used, desire did not exert a unique influence on placebo effects, but interacted with expectations to modulate placebo analgesia [34]. Reducing negative emotions also have been posited to mediate placebo effects [35, 36]. Some studies have demonstrated that induced fear of pain could weaken the magnitude of placebo analgesia [37]. However, compared with expectations and learning, these behavioral bases are still under-investigated and require further research.

### Neural responses underlying placebo and nocebo effects

Earlier studies have shown that placebo analgesia can be blocked by naloxone, indicating that the endogenous opioid and descending pain modulatory system (DPMS) play a crucial role in placebo analgesia [38]. Key regions in the DPMS originate in the cingulate cortex and prefrontal cortex (PFC) and project directly and indirectly to the periaqueductal gray (PAG), and the PAG in turn sends projections to the rostroventral medulla (RVM) and spinal cord. Recent brain imaging studies provide further support for the involvement of the DPMS in mediating placebo analgesia and nocebo hyperalgesia [39, 40]. Functional magnetic resonance imaging (fMRI) and positron emission tomography (PET) studies have shown placebo-related activity increases in brain regions including the cingulate cortex, ventromedial PFC (vmPFC), dorsolateral PFC (DLPFC), anterior insula, PAG, and RVM [39, 41–47]. Nevertheless, it is still not clear at which stage the DPMS inhibits the noxious signal input. Two participant-level meta-analyses of 20 functional neuroimaging studies in 603 participants have confirmed that placebo analgesia only has small effects on the ‘neurologic pain signature’, a machine learning-derived functional imaging correlates of pain [48], but is likely to act at the level of several brain networks beyond nociception that may be important for the emotions, decision making, and behaviors surrounding pain [49, 50]. Furthermore, the release of endogenous opioids in the DPMS could be relatively slow, so they are less likely to mediate cue-based expectations on pain, which is expected to be transient and reversible [9]. Thus, the DPMS may be just one of the mechanisms underlying placebo analgesia.

The reward system is another likely neural underpinning of placebo analgesia since symptom reduction (decreased suffering) is a special case of reward. Furthermore, the expectation is closely related to the activation of tegmental or prefrontal dopaminergic neurons that project to the dorsal and ventral striatum/nucleus accumbens (VS/NAc) [51–53]. Across studies, placebo analgesia increased fMRI responses [54, 55], opioid [44, 45, 56], and dopamine [44] activities in the NAc during pain. Importantly, the isolated brain regions (e.g., NAc and PFC) in the reward system form the mesocortical (originates from the ventral tegmental area [VTA] and projects primarily to the frontal lobe, e.g., rostral anterior cingulate cortex [rACC]) and mesolimbic pathways (originates from the VTA and projects primarily to the ventral striatum, e.g., NAc) to encode expectancy effects on pain (i.e., placebo analgesia and nocebo hyperalgesia), and to explain individual differences of the magnitudes of placebo and nocebo effects [14]. Apart from placebo analgesia, the reward system is also implicated in placebo effects in Parkinson’s disease. An early PET study showed that placebos trigger the release of endogenous dopamine in the striatum [51]. Later studies reconfirmed the involvement of dopamine release in the dorsal and ventral striatum in placebo effects in Parkinson’s disease [57].

It is still under debate whether placebo and nocebo effects are engaged in the same brain network with opposite activity directions [58]. Some studies have suggested that the DPMS and reward system might be essential for both placebo and nocebo effects [14, 44, 59–61]. They elicit opposite responses of endogenous opioid neurotransmission in the DPMS and of dopamine neurotransmission in the reward system [44]. Others suggest that placebo and nocebo effects recruit different neural circuitry and release distinct substances (e.g., cholecystokinin for nocebo effects) [62, 63]. A recent meta-analysis also showed placebo-specific concordance in the ventral striatum and nocebo-specific concordance in the posterior insula and dorsal ACC [64]. Overall, placebo and nocebo effects may be associated with both shared and distinct mechanisms/pathways.

## Harnessing placebo and nocebo effects using psychosocial, pharmacological, and neuromodulation approaches

### Psychosocial approaches

**Fig. 1:**
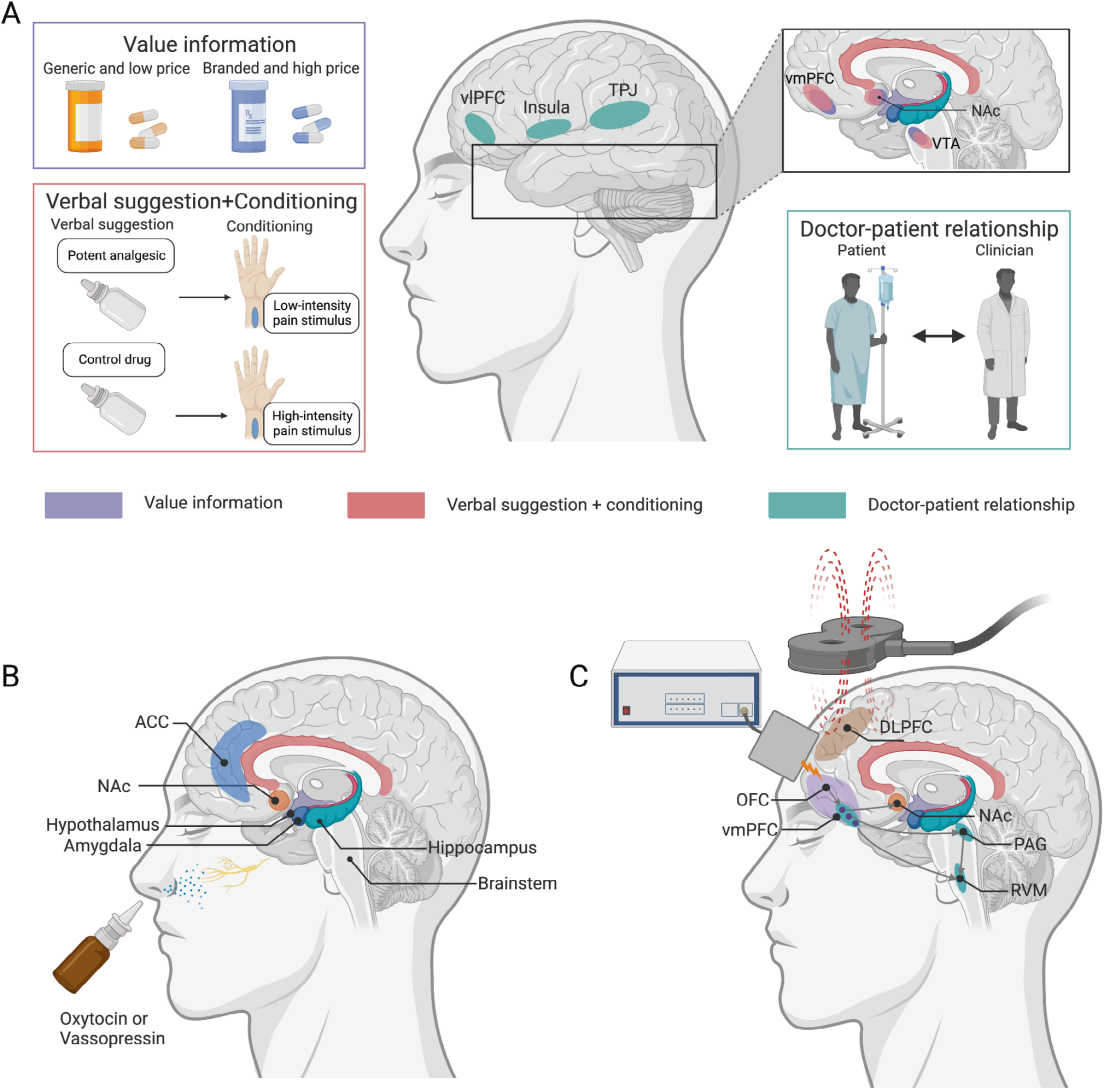
Different approaches and potential neural mechanisms to harness placebo effects. **A** Psychosocial approaches, including valuable information and enhanced conditioning (i.e., verbal suggestion precedes conditioning), modulate the reward system (e.g., ventromedial prefrontal cortex [vmPFC], nucleus accumbens [NAc], and ventral tegmental area [VTA]). Trustful doctor–patient relationships rely on the brain-to-brain coupling in the temporoparietal junction (TPJ), insula, and ventrolateral prefrontal cortex (vlPFC). **B** Intranasally administered oxytocin/vasopressin travels to the brain via olfactory and trigeminal nerve fibers and may modulate placebo-related brain activities in the anterior cingulate cortex (ACC), NAc, hypothalamus, amygdala, hippocampus, and brainstem. **C** Neuromodulational approaches including transcranial magnetic stimulation (TMS) and electrical stimulation (tES) target key regions in the prefrontal cortex (e.g., dorsolateral prefrontal cortex [DLPFC] and orbitofrontal cortex [OFC]) to modulate the reward system and descending pain modulation system (e.g., periaqueductal gray [PAG] and rostral ventromedial medulla [RVM]) to harness placebo effects.

Based on the psychological mechanisms introduced above, modulating expectations, learning, and social interactions are three major psychosocial approaches to harnessing placebo and nocebo effects (shown in Fig. 1A). Expectation manipulation can be easily achieved by altering external characteristics of placebo treatments, such as brand names (generic vs. branded) and value information (expensive vs. cheap). Compared with generic tablets (e.g., generic Ibuprofen), branded ones (e.g., Nurofen) not only have a greater efficacy but also produce fewer side effects [65]. This phenomenon may be explained by individuals’ perceiving generic drugs as less effective and of poorer quality [66]. The price tag of treatment also conveys information about its value or quality, suggesting a role of price in placebo effects [67, 68]. This was recently confirmed by a study using two placebo creams (high vs. low price), showing the higher-priced placebo treatment led to enhanced pain relief, which was associated with fMRI responses in the NAc, vmPFC, and ventral tegmental area [69]. The effect of price on placebo magnitude and brain activity also occurs in Parkinson’s disease [70]. Interestingly, higher-priced medications may also lead to an increase in perceived side effects (i.e., nocebo effects), suggesting that participants may infer that expensive medication contains a more potent and effective agent and consequently produces more side effects [71].

Although classical conditioning can induce significant placebo and nocebo effects, it is suggested that the combination of conditioning and verbal suggestion/instruction brings stronger effects [12]. Interestingly, the chronology of verbal suggestion and conditioning, as well as their congruence, influence the magnitudes of placebo and nocebo effects [72]. Participants may have stronger placebo analgesia when the verbal suggestion proceeds rather than follows conditioning, while the order of the procedures does not affect the magnitude of nocebo hyperalgesia.

Minimizing nocebo effects is of profound clinical implications. One intriguing yet thorny issue in nocebo hyperalgesia is that it seems not subject to extinction once established through classical conditioning [73, 74]. However, a recent study showed that nocebo hyperalgesia can be attenuated by classical extinction and counterconditioning with a larger trial number, where conditioned aversive cues are later paired with positive stimuli [75]. Interestingly, conditioning-induced nocebo itch can even be reversed by counterconditioning [76].

Social interaction between healthcare providers and patients typically occurs in medical treatments. In particular, the doctor-patient relationship is critical in maximizing treatment beliefs/expectations and other non-specific treatment effects, and, thus, enhancing the placebo effects and the total treatment efficacy [13, 77]. Two early studies demonstrated that a supportive doctor–patient relationship is a robust component of the placebo effect (i.e., boosts patients’ expectations towards the treatment) and can enhance therapeutic effects in large single-blind randomized clinical trials [78, 79]. A recent effort has been made to modulate and study how doctors’ expectations can be transmitted to patients and affect clinical outcomes [80]. The study highlights the importance of healthcare providers’ behavior and cognitive mindsets in affecting clinical interactions.

It is worth noting that while the behaviors of the doctor may influence the patient’s expectancy/belief, there is no guarantee that these manipulations would be effective. For instance, researchers applied a context manipulation model [78] to test if enhancing the doctor–patient relationship could increase the expectancy and treatment effect of acupuncture on chronic low back pain (cLBP). Results showed no significant differences between the high- and low-context groups in both back pain severity and expectancy scores [81]. These findings suggest gaining patients’ trust and enhancing expectancy is a complicated process, and warmness and empathy may be just two of several factors that can influence their expectations.

### Pharmacological approaches

The pharmacological approaches have gained interest due to their potential in modulating key psychosocial components of placebo and nocebo effects (Fig. 1B).

Oxytocin is a peptide hormone and neuropeptide that plays a role in empathy, trust, and social learning. Recently, studies have shown oxytocin can promote social cognition and learning [82, 83], enhance empathy levels [84], and reduce stress and anxiety [85]. Thus, some researchers hypothesized oxytocin may enhance the placebo effect. An earlier study found oxytocin can enhance verbally induced placebo analgesia in males [86]. Nevertheless, several recent studies report insignificant modulatory effects of oxytocin on placebo analgesia and nocebo hyperalgesia [87–89]. Therefore, experimental evidence supporting the role of oxytocin for placebo and nocebo effects is mixed, at least in the field of pain.

Vasopressin is another potential candidate to modulate placebo effects. The brain distribution of oxytocin receptors overlaps with those of arginine vasopressin, and studies suggest that vasopressin can regulate conciliatory behaviors and social communication in females [90, 91]. A previous study found that vasopressin agonizts boosted placebo effects in women but had no effect in men [92].

Two aspects of these pharmacological studies on placebo/nocebo effects are noteworthy. One is that most of them recruited healthy participants, not patients [93]. As a result, it remains an open question whether and how these findings can be translated to clinical application. The other is that they induced placebo or nocebo effects mainly by verbal instructions. Neurotransmitters like oxytocin and vasopressin influence the outcome of conditioning [94, 95], so it is of interest to investigate whether conditioning-induced placebo and nocebo effects can be pharmacologically modulated.

### Neuromodulation approaches

The past decade has witnessed a growing interest (or rediscovery, as the concept of brain stimulation has existed for over one hundred years) in the modulation of human behavior and cognition by noninvasive brain stimulation (NIBS). These methods allow researchers non-invasively alter neural activity/excitability (enhancing or inhibiting) to affect behaviors [96]. Two NIBS methods have emerged in both basic and clinical contexts: transcranial magnetic stimulation (TMS), which sends pulses to increase cortical excitability due to long-term potentiation or to inhibit cortical excitability due to long-term depression, and transcranial electrical stimulation, which passes low-intensity electrical currents through the cortex and de- or hyperpolarizes neuronal membrane potentials to alter cortical excitability.

Given the important role of the brain in placebo and nocebo effects, it is a natural next step to apply NIBS tools to modulate the placebo effect as well as investigate the mechanism of placebo/nocebo effects to elucidate the causal role of certain brain regions (Fig. 1C). For instance, across neuroimaging studies, the most consistent placebo-related brain responses in the PFC are observed in the DLPFC, vlPFC, and vmPFC (including the rACC and pregenual anterior cingulate cortex, and orbitofrontal cortex [OFC]) [14, 18, 19, 40, 41, 55, 61, 67, 97]. The converging findings in the PFC bring valuable targets for harnessing placebo and nocebo effects through directly modulating brain responses in these precise locations.

In an early study, investigators found that low-frequency repetitive TMS (rTMS) at the DLPFC (aiming to transiently disrupt left and right DLPFC function) could block expectation-induced placebo analgesia as measured by pain threshold and tolerance increases [98]. The following study investigated the modulation effect of single-session transcranial direct current stimulation (tDCS) at the right DLPFC and showed that placebo and nocebo effects could only be observed in participants who received anodal tDCS (to enhance neuronal excitability) but not in those who received cathodal tDCS (to inhibit neuronal excitability) [99]. In a more recent study, we found that multi-session (three sessions) repeated tDCS at the left OFC and right DLPFC could boost placebo and blunt nocebo effects, as well as modulate brain activity and connectivity associated with placebo analgesia and nocebo hyperalgesia, respectively [97].

These findings together not only demonstrate the feasibility of harnessing placebo and nocebo effects through changing brain excitability with NIBS but also suggest how experimentally altered neural activity causally affects placebo and nocebo effects. Nevertheless, caution must be exercised when applying NIBS to modulate specific brain areas or networks, because the NIBS-induced effects are more complex than their computational models and these effects are subject to stimulation protocols (e.g., current intensity [100], stimulation duration [101]) and individual’s brain characteristics (e.g., the orientation of the axons in relation to the current flow [102], baseline brain state [103]).

A noticeable limitation of NIBS is that the targeted areas are generally limited to cortical regions due to low penetrance [96]. On the other hand, abundant subcortical areas (e.g., the NAc and PAG) and complicated brain networks are involved in placebo/nocebo effects. Most of them cannot be directly modulated via NIBS. Deep brain stimulation can reach subcortical areas but are undesirable for many patients due to its invasiveness. Two strategies may be adopted to partially overcome this limitation of NIBS. One is to target multiple brain regions simultaneously; the other is to modulate areas that are hubs of placebo-related brain networks or exhibit strong connectivity with subcortical areas. Future studies may test the effectiveness of these strategies in the context of placebo and nocebo effects.

## Translating basic research findings into clinical treatment settings

### General principles in clinical practice

Translating basic research findings of placebo and nocebo effects into clinical treatment settings is a high-stake issue. To maximize the salubrious placebo effects as well as minimize the detrimental nocebo effects in clinical care, some generally agreed-upon guidelines for utilizing placebo and nocebo effects in clinical practice have been recently suggested [104]. Based on these guidelines, general principles that health care providers can use to elicit placebo effects and reduce nocebo effects include:

- The modulating expectation is always helpful to induce placebo effects in medical practice. Healthcare providers may point out directly that a drug or treatment is effective if its efficacy has already been proven. Informally explaining the mechanisms of treatments and placebo effects may also be of benefit. Such knowledge promotes trust in the treatment and boosts positive expectations toward efficacy.
- Increasing knowledge mitigates nocebo effects. Misattribution of accidental experiences or preexisting symptoms to treatments will amplify nocebo effects [105]. Demystifying nocebo effects by increasing knowledge is thus a crucial step to preemptively nullify misattribution. Indeed, recent studies have shown that informing participants with weekly headaches about nocebo effects reduced the nocebo side effects they experienced [106], and providing timing information minimized nocebo effects because they often occurred when individuals expected them to occur [107].
- Improving the communication style to build a supportive relationship between patients and physicians triggers placebo effects [77]. On the other hand, a cold, indifferent, impatient, or hostile relationship induces nocebo effects [108]. Nevertheless, enhancing expectancy and gaining patients’ trust is a complicated process. The supportive relationship may only work for some individuals. In addition, “exaggerated” positive information and “over” supportive relationships should be used with caution to avoid ethical concerns.

### Applying the learning model in clinical treatment

Experimental studies have demonstrated that a conditioning-like manipulation model can produce greater placebo effects compared to verbal suggestion/instruction alone [109]. In addition, this model can also enhance the effect of active treatments on experimental pain [54, 110]. Nevertheless, few studies have applied the expectancy manipulation model in longitudinal treatment in patient populations due to the difficulty in modulating chronic pain intensity compared to experimental pain. To overcome this challenge, investigators have applied an expectancy manipulation model using experimental heat pain to enhance subjects’ expectation of acupuncture analgesia (on heat pain), and then confirmed that this enhanced expectation improved the treatment effect of acupuncture on chronic pain caused by knee osteoarthritis (KOA) [111]. This study demonstrated the feasibility of applying the expectancy manipulation model in clinical settings, which may shed light on improving treatment effects.

Observational learning and operant conditioning can also be considered when clinicians interact with patients to induce placebo effects [23, 30]. Arguably, they can be more easily applied than classical conditioning in clinical treatment, since observational conditioning involves only indirect information about treatment effectiveness from other individuals and operant conditioning requires only appropriate reinforcement like a verbal reward. A recent clinical trial has proved the role of observational learning in enhancing placebo analgesia in cLBP patients [112]. However, operant conditioning has only recently been put forth as a new mechanism of placebo effects [30], and no clinical studies have empirically examined its ability to produce placebo effects in patients. Further research is thus in need to test the clinical applicability of operant learning.

### Variability of placebo effects

It has long been acknowledged that placebo effects exhibit large individual variabilities [113]. Clinical application of placebo effects has to account for these variabilities. Demographical, psychological, and biological factors have all been linked to individual variabilities in placebo effects.

Sex and race matter for placebo effects. Females and white populations seem to experience larger placebo effects [114, 115]. Another set of important predictors of placebo effects is psychological factors, e.g., expectation, trait optimism, desire for control [116], emotional distress, and maladaptive cognitive appraisals of pain [117]. In clinical studies, patients susceptible to a placebo effect can be identified by assessing pretreatment positive and negative expectations [118, 119], and prior therapeutic experience via conditioning [120]. Brain activity or brain structures have also been used to predict placebo effects. Stronger placebo effects have been associated with a more efficient reward system (e.g., NAc responses to reward cues [121], gray matter densities of the NAc and PFC [14, 122], regional homogeneity of NAc [123]) and frontoparietal network functional connectivity [21]. Importantly, using machine learning and fMRI, studies were able to identify placebo responders and predict the magnitude of placebo effects in patients with KOA [124], major depression [125], and cLBP [81, 126].

### Placebo effects in patients and healthy individuals

Patients and healthy individuals differ considerably in many ways. For example, chronic pain patients typically suffer from anxiety and depression [127]. More importantly, the neural underpinnings of placebo effects are impaired in some diseases like chronic back pain [128] and fibromyalgia [129]. A crucial issue is then whether findings based on healthy individuals can be generalized to patients. A meta-analysis has shown that the magnitude of placebo analgesia in studies with healthy participants was smaller than in studies with patients, but the difference was not statistically significant [130]. Recent studies directly comparing chronic pain patients and healthy controls also found that the magnitude of placebo analgesia was comparable between healthy controls and fibromyalgia, osteoarthritis, and chronic orofacial pain patients [120, 131, 132]. These findings suggest that chronic pain may not significantly affect patients’ susceptibility to placebo effects, even though it may impair neural pathways key to placebo analgesia. One explanation is that these impaired areas and pathways are not necessary for placebo analgesia, since multiple distributed neural networks are involved in placebo effects. However, it still remains an open question whether disease impairs the ability of psychosocial, pharmacological, and neuromodulation approaches to modulate placebo and nocebo effects. Due to shared psychological and neural mechanisms, it is reasonable to assume that placebo and nocebo effects can also be modulated similarly in patients and healthy people. Nevertheless, future research needs to directly test the feasibility of boosting placebo effects and blunting nocebo effects in patient populations to harness these effects in clinical settings.

### Considerations in clinical application

When applying placebo/nocebo effects, one must be sensitive to the clinical issues involved. A placebo lies not in the drug or procedure itself, but in the patient’s own mind (or brain). Persuading the patients that a placebo treatment works may involve deception and violation of their autonomy. One arguably less concerning approach for harnessing placebo/nocebo effects is to adopt open-label placebo treatments, in which the inertness of the treatment and the efficacy of placebos are revealed explicitly. Randomized clinical trials of open-label placebos in different conditions, including patients with irritable bowel syndrome [133], cLBP [134], cancer-related fatigue [135], and episodic migraine [136], have demonstrated the therapeutic efficacy of open-label placebos. A recent meta-analysis of 13 open-label placebo clinical trials found a significant overall effect of open-label placebos as compared to no treatment but also cautioned that current studies were still immature [137].

One potential issue for open-label placebo treatment is that the power of the verbal suggestion (informing the participants that studies have shown placebos can also produce treatment effect) may fade with wide application of the open-label placebo treatment, as open-label placebos may tend to be less effective than real medication. One solution to this issue is to combine active treatments with placebos [138]. Exploiting the power of placebo effects to boost, not replace the efficacy of active interventions, could induce fewer clinical concerns. Dose-extending placebos may raise fewer clinical problems than pure placebos. By interspersing placebos between real medications, dose-extending placebos not only induce placebo effects, but also have many practical advantages such as cutting medication intake, reducing medication dependence, and likely decreasing financial costs for patients [139]. Since potent treatments are also used, dose-extending placebos are presumably less clinically problematic. Combining open-label placebos with dose-extending placebos (i.e., open-label dose-extending placebos) further reduces clinical concerns. Admittedly, even this combination of two clinically less concerning placebos is not perfect. However, surveys have shown that a fair proportion of health providers prescribe placebos in real clinical settings [140, 141]. Since placebo and nocebo effects are almost inevitable, the real question is not whether clinicians should apply these effects, but how they can make use of current findings to better apply placebo and nocebo effects while keeping ethical considerations in mind.

## Open questions and conclusion

In this review, we surveyed important advances in understanding behavioral and neural mechanisms of placebo and nocebo effects and employing psychosocial, pharmacological, and neuromodulation approaches to harness these effects and discussed the challenges to applying these findings to medical practice. The mechanistic heterogeneity of placebo and nocebo effects in different domains is worth noting. Up till now, most placebo and nocebo studies have come from pain. Nevertheless, general psychological (e.g., conditioning, expectations) and brain mechanisms (e.g., the DLPFC, reward system) identified from pain-related studies may be not specific and could be shared with placebo effects across other domains/symptoms [27].

Using psychosocial approaches to modulate placebo and nocebo effects might be low-cost and easy to do in clinical applications. However, it is noteworthy that psychosocial approaches may be dependent on an individual’s different preferences or personality in gaining expectancy/belief (e.g., someone may prefer warm conversations with the doctor, while others may prefer less conversation), and therefore the modulatory effects may vary considerably across individuals. How to build a trustful doctor–patient relationship in the clinical setting should be carefully characterized and studied based on different populations rather than a general style/suggestion.

Although promising, findings concerning the efficacy of pharmacological approaches are somewhat mixed [87]. Current efforts mainly focus on modulating pain-related placebo and nocebo effects. It is worth testing if oxytocin and vasopressin can modulate other placebo and nocebo effects. For example, since oxytocin can promote social trust in humans [142], its role in modulating a trustful doctor-patient relationship should be tested in future studies.

Ideally, it is desirable to simultaneously enhance placebo and inhibit nocebo effects by changing brain excitability. As mentioned above, placebo and nocebo effects have both shared and distinct mechanisms. Achieving these two aims at once would thus be challenging. Indeed, an effective brain target for simultaneously harnessing these two effects is still inconclusive and needs experimental validation. However, it is worth trying to target with NIBS presumably shared brain areas (e.g., the DPMS and reward system) underlying placebo analgesia and nocebo hyperalgesia [44].

In summary, placebo and nocebo effects are powerful, pervasive, and common in cognitive neuroscience and clinical practice. Moving from native observation to experimental mechanistic manipulation, and finally utilizing the effects wisely in clinical practice may lead to the improvement of therapeutic outcomes and minimization of unintended exacerbation of symptoms.
